# 

**Published:** 1897-07

**Authors:** 


### CONTENTS.

ORIGINAL ARTICLES: PAGE
- Annual Address of the President of the Alumni Association of the American Veterinary College. By L. H. HOWARD, D.V.S. . . . . 423
- Involuntary Twitching of the Head Relieved by Trifacial Neurotomy. By W. L. WILLIAMS, V.S. . . . . 426
- Contribution to the Study of the Horse-chestnut. By J. L. CLARK, V.S. . . . 428
- The Inspection of Dairies. By GEORGE W. MCGUIRE . . . . 430
- Tuberculosis. By J. J. OBERST, M D.C. . . . . 436
- Azoturia. By JOHN R. HART, V.M D. . . . . 440
- A Case of Acromegaly in a Dog. By R. H. CUNNINGHAM, M.D. . . . 444

ABSTRACTS FROM FOREIGN JOURNALS:
- Some Statistics in Regard to Tuberculosis . . . . 449
- Sand in the Stomach of Ruminants . . . . 450

REPORTS OF CASES:
- Traumatic Pericarditis in a Cow; Recovery. By W. L. WILLIAMS, V.S. . . . 451
- Luxation of the Metacarpo-pharyngeal Articulation in a Horse. By W. L. WILLIAMS, V.S. . . . . 452
- A Post-mortem Revealing Two Spleens. By JAMES M. RICHARDSON . . . 452

EDITORIALS:
- Our Broadest Future is Yet Before Us . . . . 454
- To Our Subscribers . . . . 455
- On to Nashville . . . . 456
- Sure to Have Support . . . . 457
- Move Right Along in this Line . . . . 457

BOOK REVIEWS . . . . 458

COMMENCEMENT EXERCISES:
- University of Pennsylvania—Cornell University . . . . 461

AMONG THE COLLEGES . . . . 463

BOARDS OF EXAMINERS:
- Examination for City Veterinarian, Philadelphia—Pennsylvania State Board—Virginia State Board—Maryland Board . . . . 464

SOCIETY PROCEEDINGS:
- On to Nashville—Chicago Veterinary Society: Report on the Relations of the Veterinary Profession to the Department of Police—New York County—Oneida County—Schuylkill Valley—New Jersey and Vicinity . . . . 466

PERSONAL . . . . 471

